# Supplementary material for: TAK1 is a key regulator of oncogenic signaling and differentiation blockade in rhabdomyosarcoma
Source: Oncogene. 2026 Apr 6;45(18):1714–28. doi: 10.1038/s41388-026-03767-z (PMC13136027; doi:10.1038/s41388-026-03767-z)
Supplement: Supplementary file 1 — Supplemental File [file 41388_2026_3767_MOESM1_ESM.pdf]

Supplemental data file

TAK1 is a key regulator of oncogenic signaling and differentiation blockade in rhabdomyosarcoma

Anh Tuan Vuong, Aniket S. Joshi, Anirban Roy, Kavya Mathukumalli, Phuong T. Ho, Raksha Bhat, Meiricris Tomaz da Silva, Tagari Samanta, Meghana V. Trivedi, Bin Guo, Benny A. Kaipparettu, and Ashok Kumar

This file contains **Figures S1-S6** and **Table S1 and S2**.

Supplemental Figures and Legends

FIGURE S1

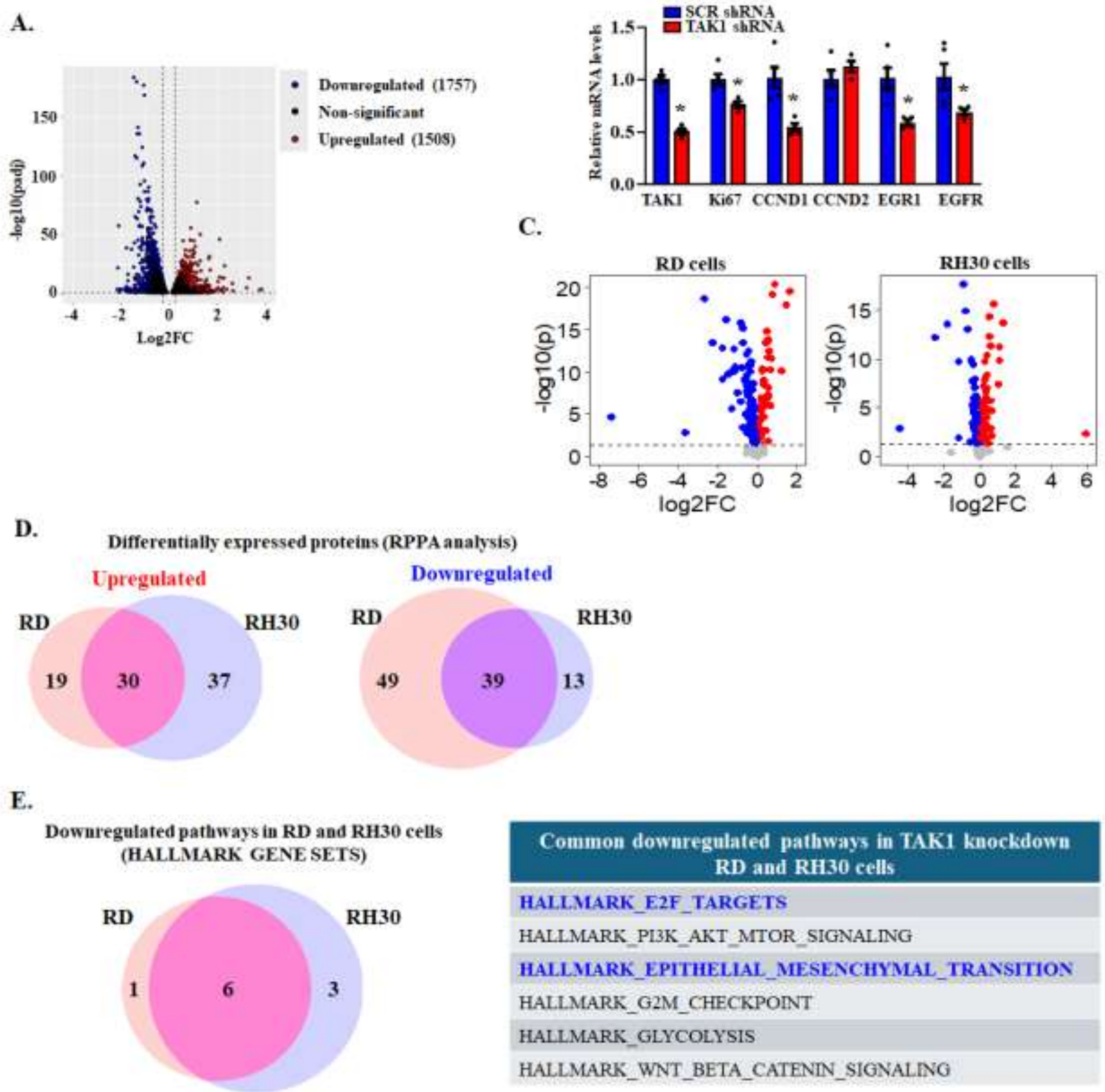

FIGURE S1. Analyis of RNA-Seq and RPPA dataset. (A) Volcano plot from RNA-seq analysis of TAK1 knockdown versus control RD cells illustrating down-regulated (blue dots) and up-regulated (red dots) genes with a threshold of $\log_2 FC \geq |0.5|$ and adjusted *P*-value ≤ 0.05. **(B)** Relative mRNA levels of TAK1, KI67, CCND1, CCND2, EGR1, and EGFR in control and TAK1 shRNA expressing RD cells. n=5 biological replicates in each group. *p<0.05, values signficantly different from scarmbled (SCR) shRNA-expressing RD cells by unpaired two-tailed t-test. **(C)** Volcano plots of RPPA analysis of TAK1 knockdown RD and RH30 cells versus corresponding control showing down-regulated (blue dots) and up-regulated (red dots) proteins

with a threshold of $log_2FC > 0$ and $log_2FC < 0$ with *P*-value < 0.05. **(D)** Vein diagram showing distinct and commonly upregulated and downregulated proteins in TAK1 knockdown RD and RH30 cells. **(E)** Pathway enrichment analysis using Hallmark gene sets in RPPA dataset show common downregulated pathways in TAK1 knockdown RD and RH30 cells.

FIGURE S2

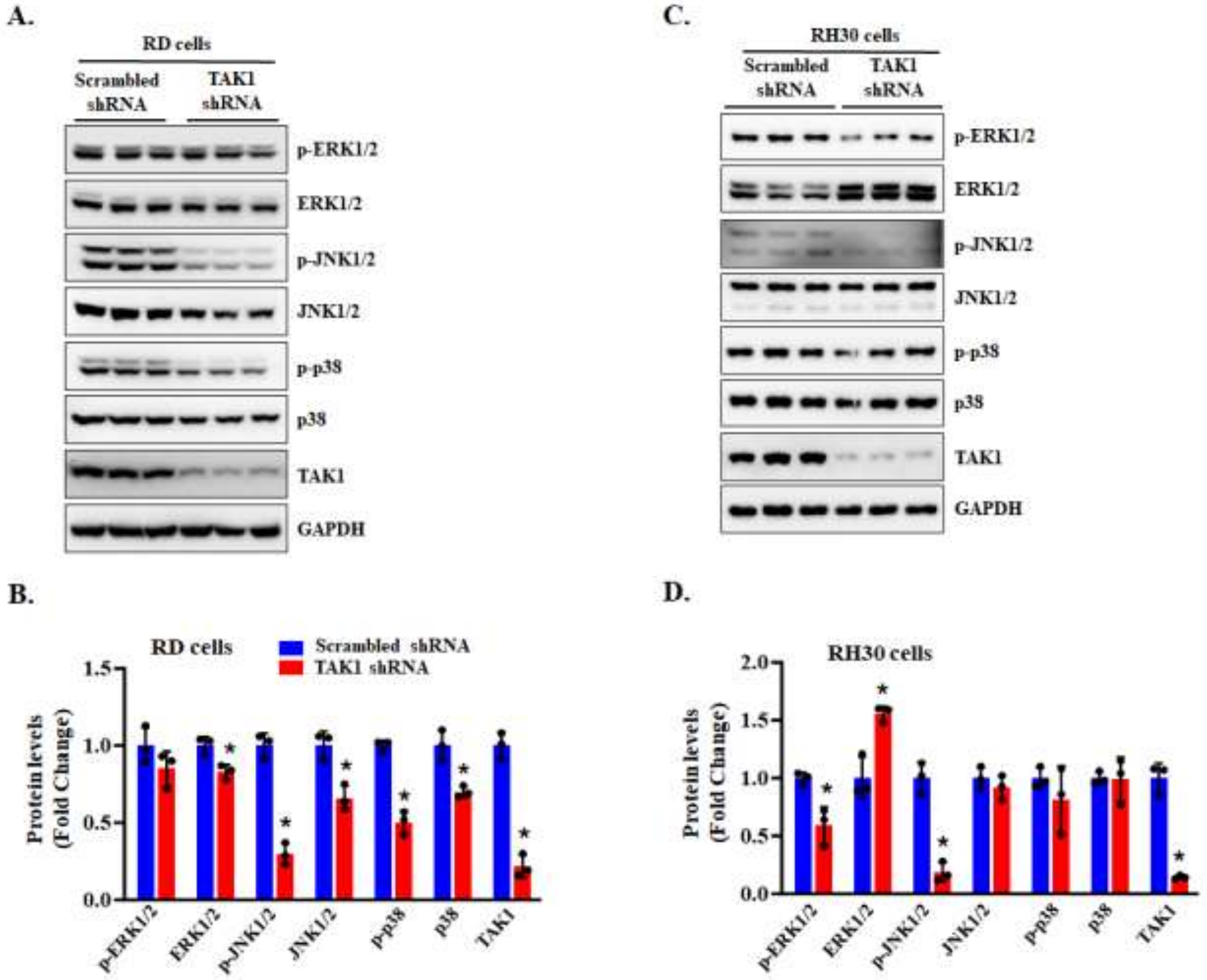

FIGURE S2. Effect of TAK1 knockdown on the phosphorylation of MAPKs in RMS cells. (**A**) Immunoblots and **(B)** densitometry analysis demonstrating the levels of phosphorylated and total ERK1/2, JNK1/2, and p38 protein and total TAK1 protein in control and TAK1 knockdown RD cells. (**C**) Immunoblots, and **(D)** densitometry analysis demonstrating the levels of phosphorylated and total ERK1/2, JNK1/2, and p38 protein and total TAK1 protein in control and TAK1 knockdown RH30 cells. n = 3 biological replicates in each group. Data are presented as mean ± SD. *$p<0.05$ from corresponding cultures expressing scrambled shRNA by unpaired two-tailed t-test.

FIGURE S3

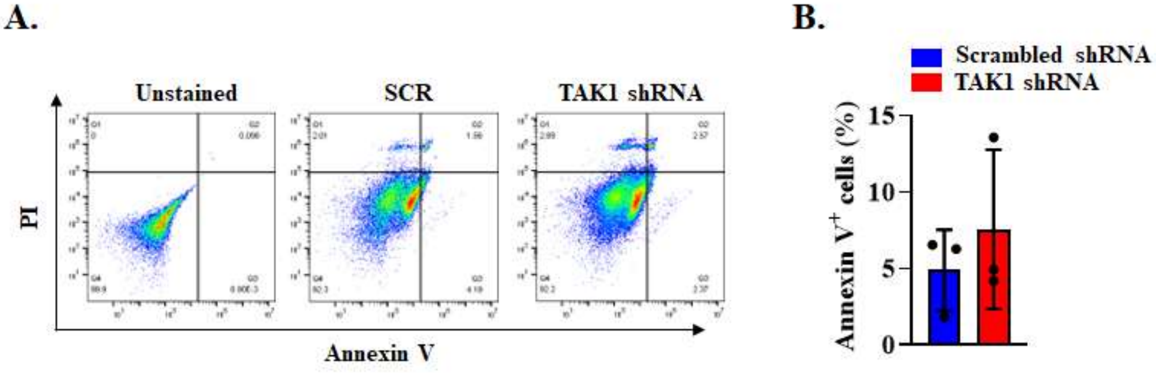

FIGURE S3. Effect of TAK1 knockdown on the survival of human myoblasts (HM). (A) Representative scatter plots of FACS-based analysis demonstrate the Annexin V-positive cells amongst control and TAK1 knockdown HM. **(B)** Quantification of Annexin V^+ cells in control and TAK1 knockdown HM measured by FACS analysis. n = 3 biological replicates in each group. Data are presented as mean ± SD. No statistically significant difference was observed between control and TAK1 knockdown cultures by unpaired two-tailed t-test.

FIGURE S4

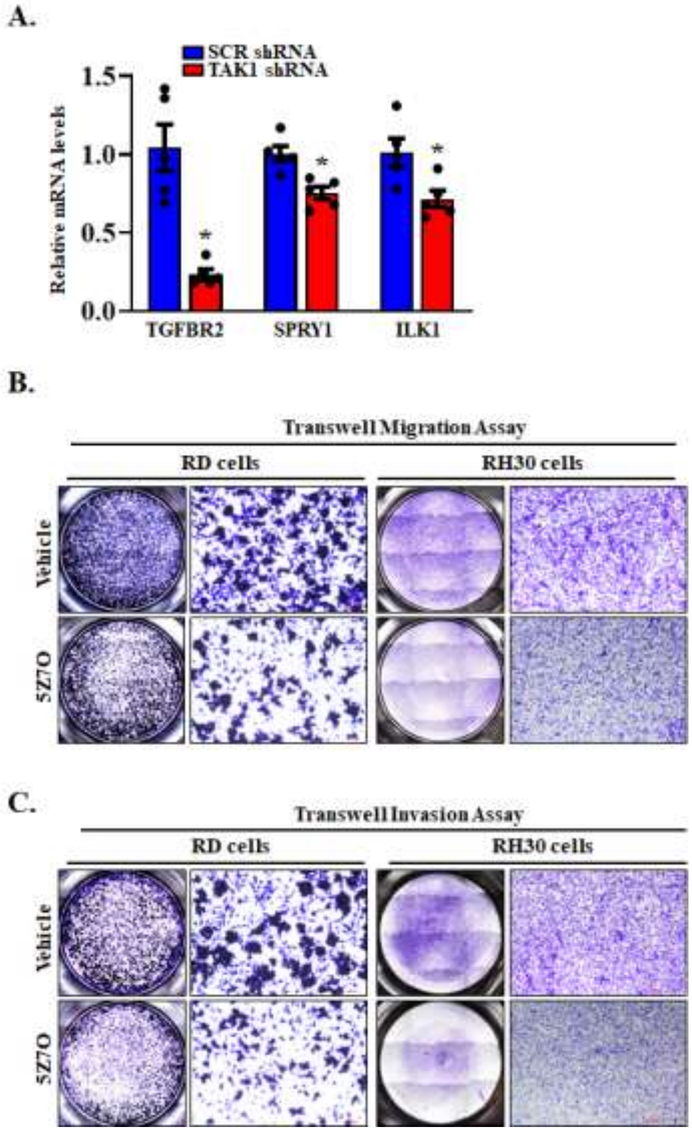

FIGURE S4. TAK1 knockdown inhibits invasion and migration of RMS cells. (A) Relative mRNA levels of TGFBR2, SPRY1, and ILK1 in control and TAK1 shRNA expressing RD cells. n=4 biological replicates in each group. *p<0.05, values signficantly different from scarmbled (SCR) shRNA-expressing RD cells by unpaired two-tailed t-test. **(B)** Representative images of vehicle and 2µM 5Z7O-treated RD and RH30 cells in transwell migration assay. Scale bars, 50 µm. **(C)** Representative images of vehicle and 2µM 5Z7O-treated RD and RH30 cells in transwell invasion assay. Scale bars, 50 µm.

FIGURE S5

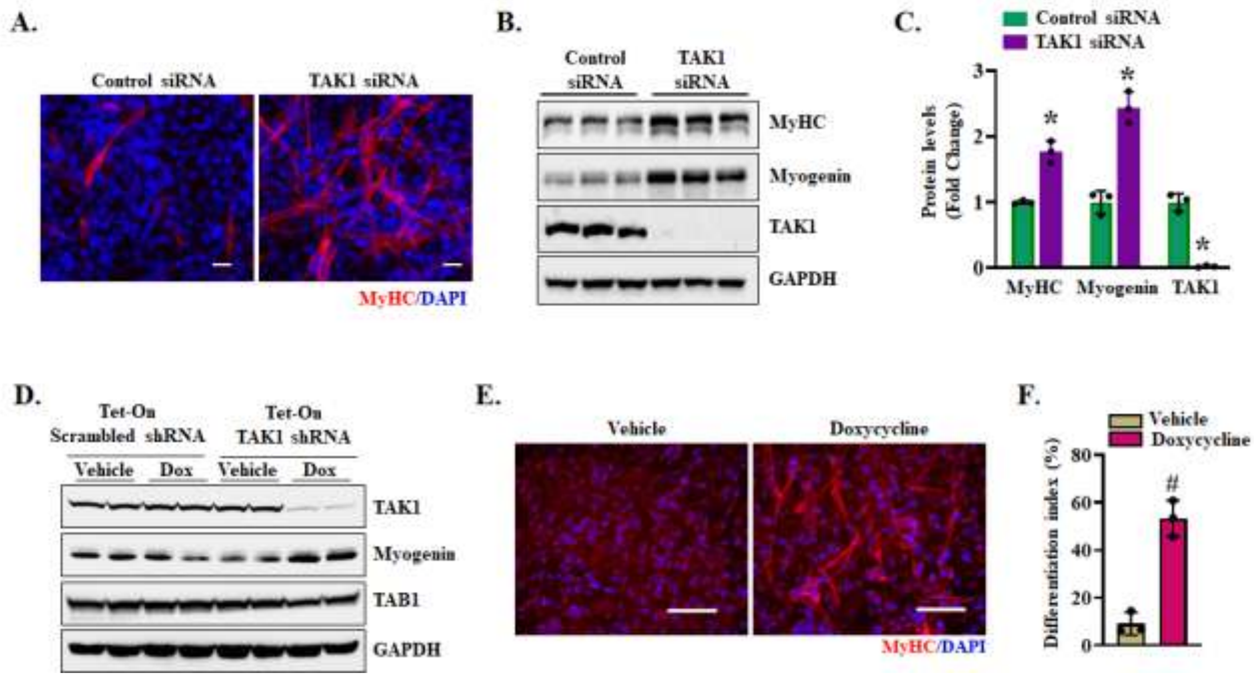

FIGURE S5. Effect of inhibition of TAK1 in myogenic differentiation of RD cells. (A) Representative photographs of control and TAK1 siRNA transfected RD cultures after immunostaining for MyHC protein. Nuclei were counterstained with DAPI. Scale bar: 20 µm. **(B)** Immunoblots and **(C)** densitometry analysis demonstrating the levels of MyHC, myogenin, TAK1, and GAPDH protein in RD cell cultures transfected with control or TAK1 siRNA. n=3 biological replicates in each group. Results are presented as mean ± SD. *$p<0.05$, values significantly different from corresponding cultures transfected with control siRNA. **(D)** Immunoblots presented here demonstrate the levels of TAK1, myogenin, TAB1, and GAPDH in RD cell cultures transduced with lentiviral particles expressing Tet-On scrambled shRNA or Tet-On TAK1 shRNA after treatment with vehicle alone or doxycycline. **(E)** Representative images of RD cell cultures expressing Tet-On TAK1 shRNA after treatment with vehicle alone or doxycycline followed by immunostaining for MyHC protein. Nuclei were stained with DAPI. Scale bar: 100 µm. **(F)** Quantification of differentiation index in control and inducible TAK1 knockdown RD cultures. n = 3 biological replicates in each group. Data are presented as mean ± SD. $^{\#}p<0.05$, values significantly different from cultures treated with vehicle alone by unpaired two-tailed t-test.

FIGURE S6

Figure 1C.

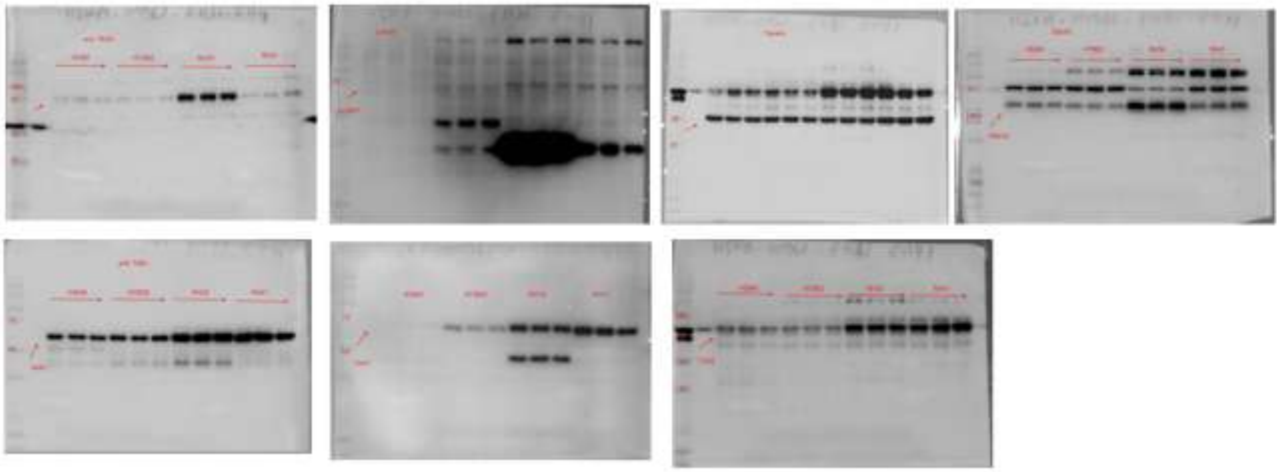

Figure 1E.

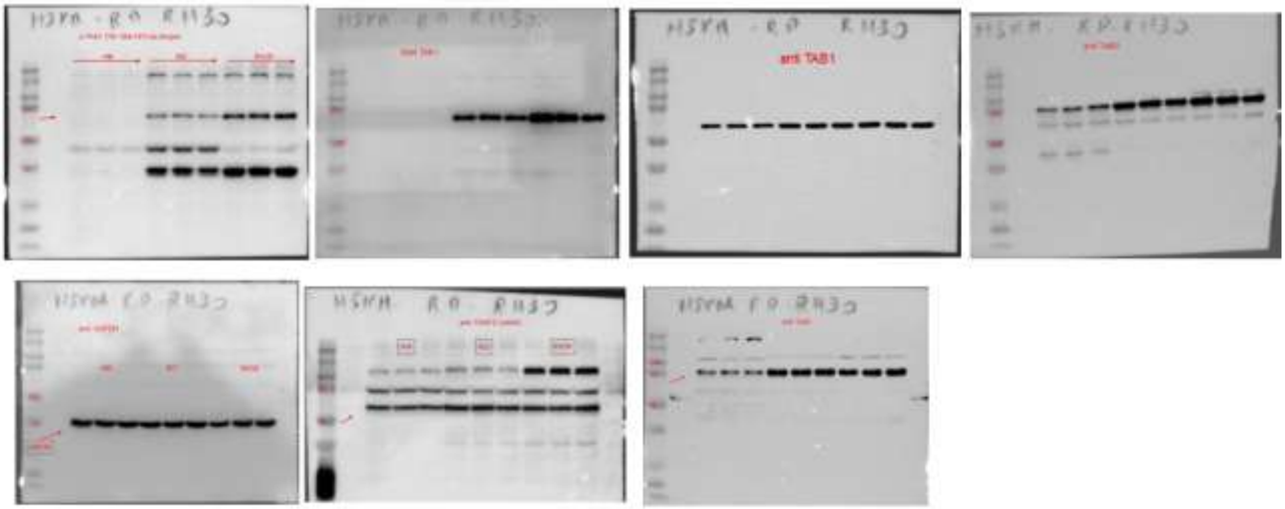

Figure 2A.

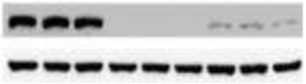

FIGURE S6 (Continuation)

Figure 5D.

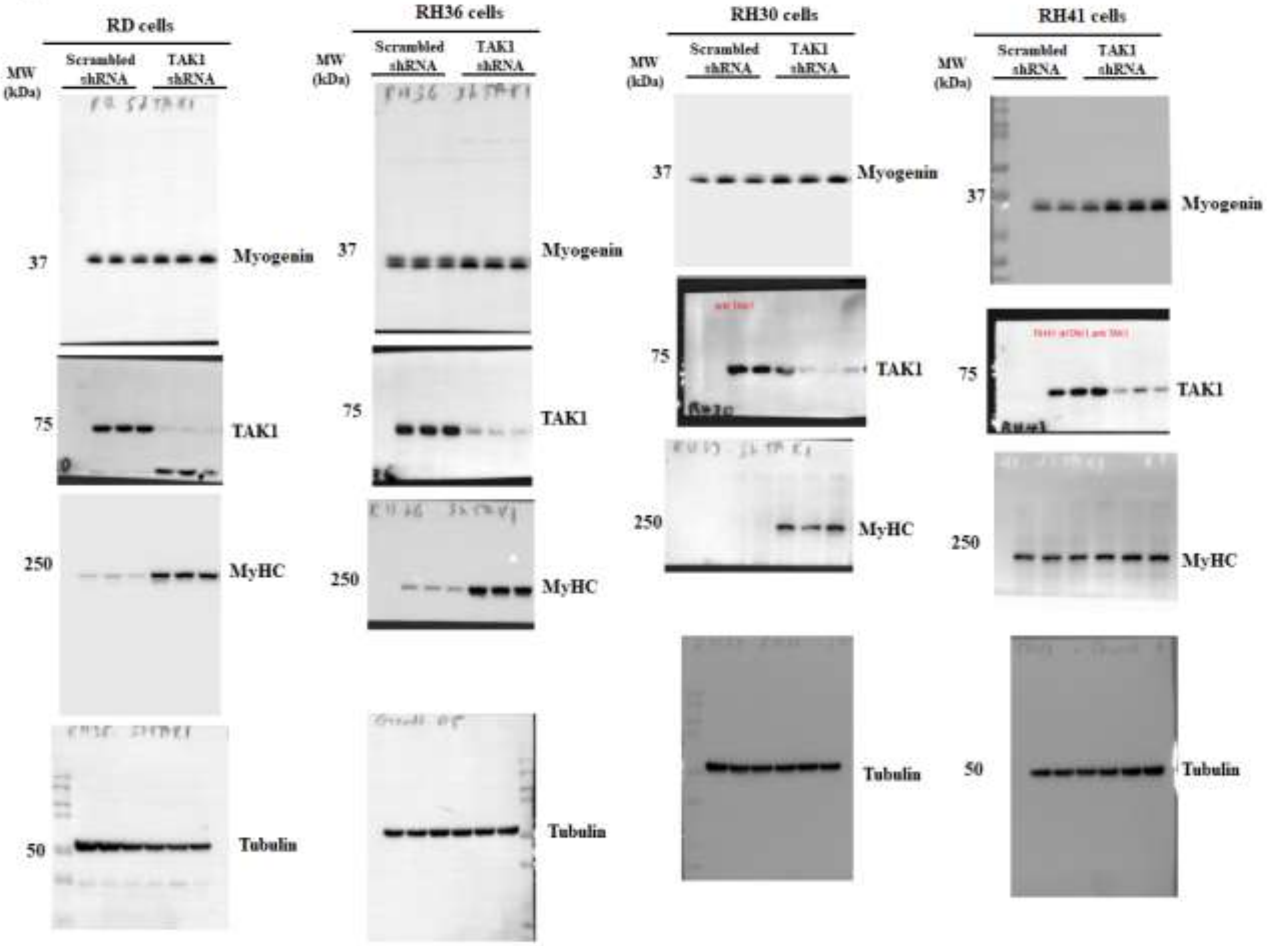

Figure 5H.

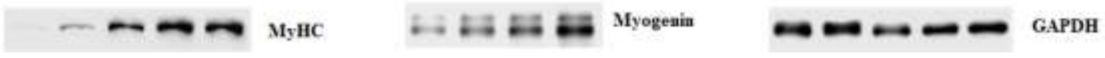

FIGURE S6 (Continuation)

Figure 6C.

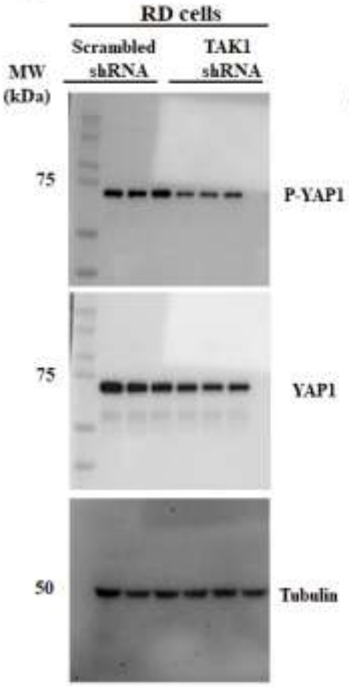

Figure 6E.

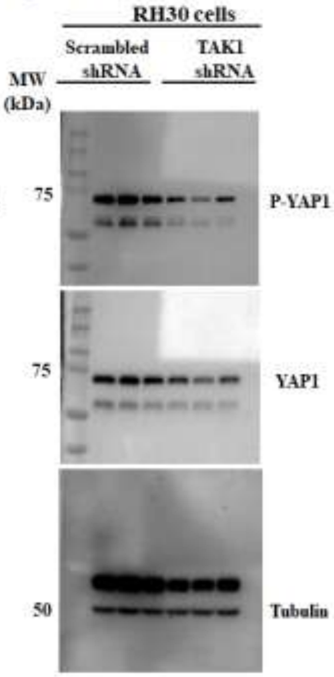

Figure 6G.

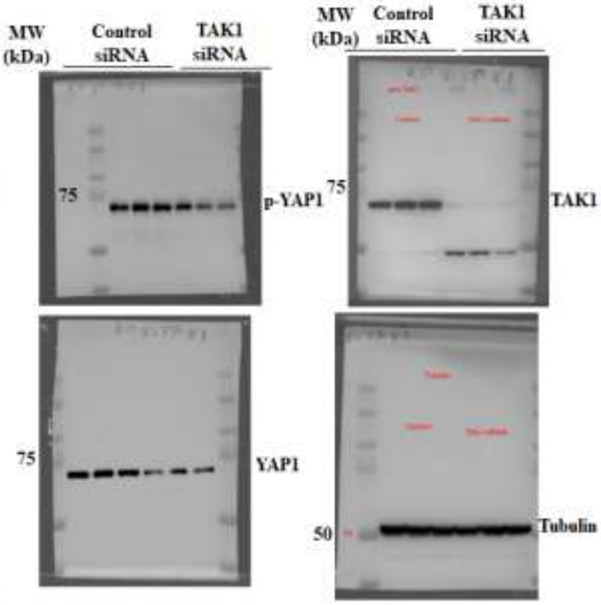

Figure 6K.

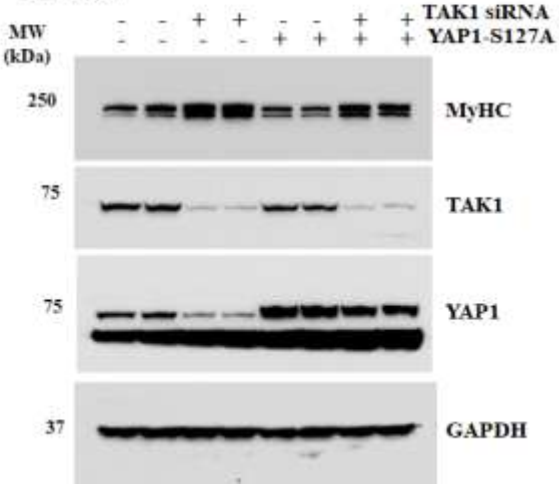

Figure 7K.

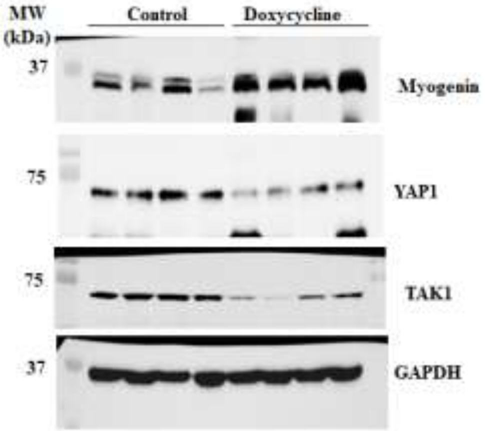

FIGURE S6 (Continuation)

Figure S2A.

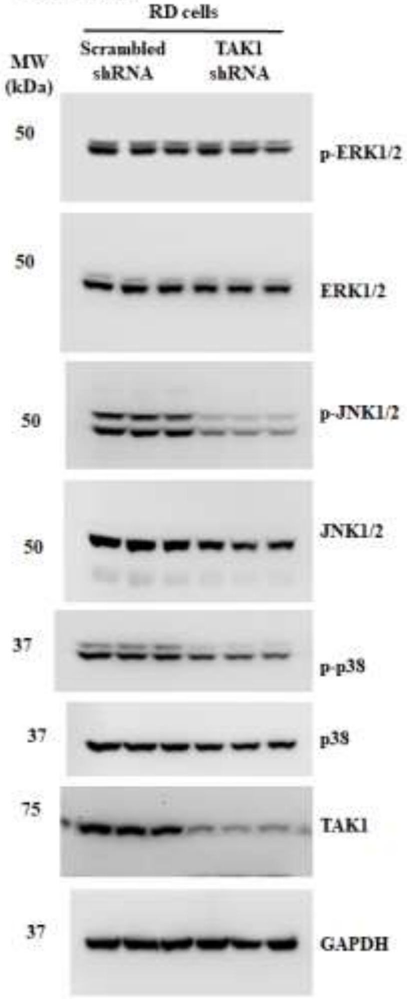

Figure S2C.

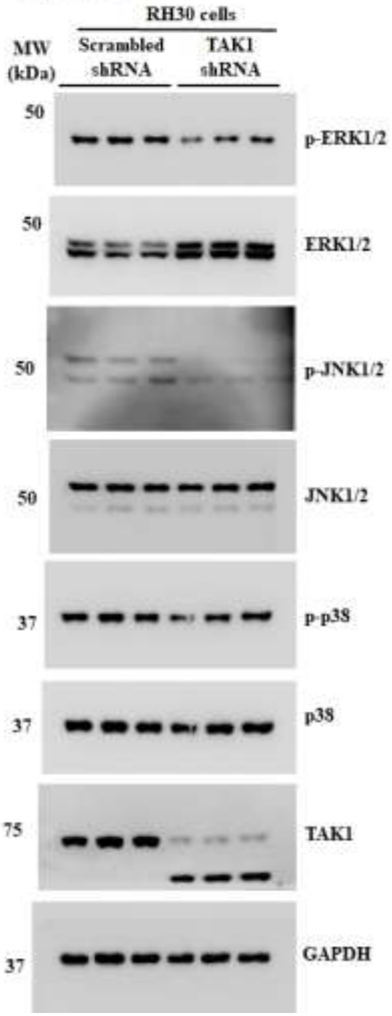

Figure S5B.

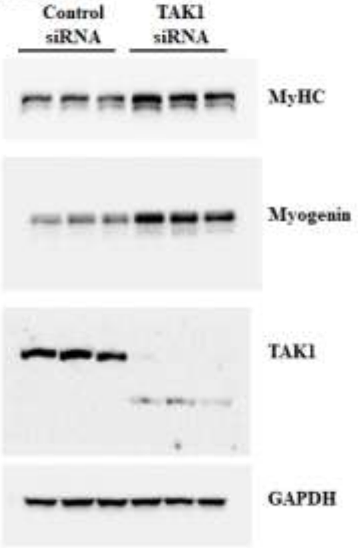

FIGURE S6 (Continuation)

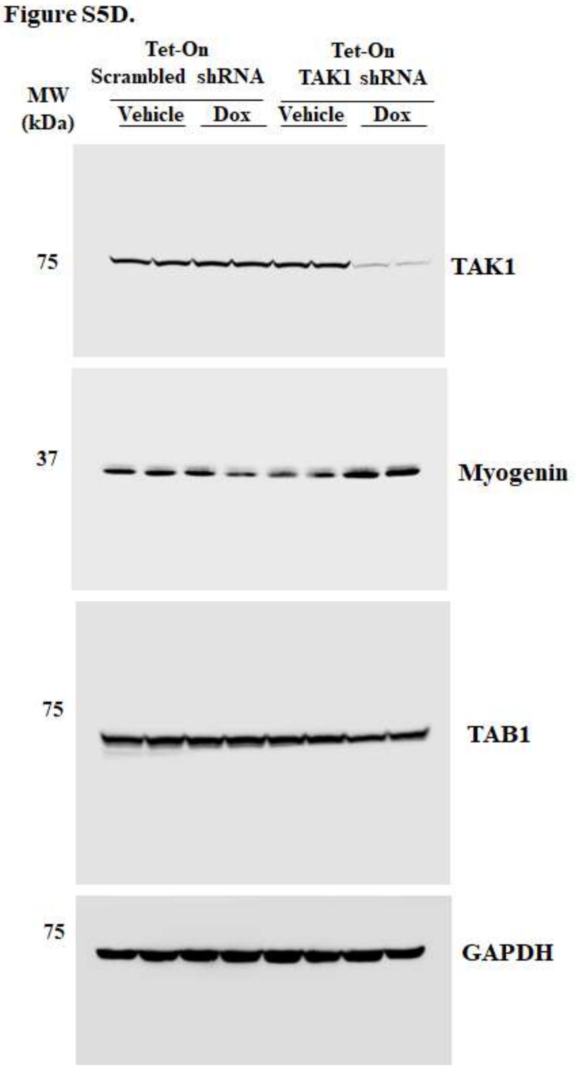

FIGURE S6. Uncropped western blot images. Uncropped immunoblot images used in the main figures and supplemental results.

Table S1. The list of antibodies used in various experiments.

Antibody	Dilution	Source	Identifier
Rabbit-anti-p-TAK1 (Thr184/187)	1:1000 (WB)	Invitrogen	# MA5-15073
Rabbit-anti-TAK1	1:1000 (WB)	Cell Signaling Technology	# 5206
Rabbit-anti-TAB1	1:1000 (WB)	Cell Signaling Technology	# 3226
Rabbit-anti-TAB2	1:1000 (WB)	Cell Signaling Technology	# 3745
Rabbit-anti-TAB3	1:1000 (WB)	Cell Signaling Technology	# 14241
Rabbit-anti-TRAF6	1:1000 (WB)	Cell Signaling Technology	# 67591
Mouse-anti-Myosin heavy chain	1:500 (WB)/1:50 (IF)	DSHB	# MF20
Mouse-anti-Myogenin	1:500 (WB)/1:50 (IF)	Invitrogen	# MA5-11486
Rabbit-anti-p-YAP1	1:1000 (WB)	Cell Signaling Technology	# 4911
Rabbit-anti-YAP1	1:1000 (WB)	Cell Signaling Technology	# 14074
Rabbit-anti-p-ERK1/2	1:1000 (WB)	Cell Signaling Technology	# 9101
Rabbit-anti-ERK1/2	1:1000 (WB)	Cell Signaling Technology	# 9102
Rabbit-anti-p-JNK1/2	1:1000 (WB)	Cell Signaling Technology	# 9251
Rabbit-anti-JNK1/2	1:1000 (WB)	Cell Signaling Technology	# 9252
Rabbit-anti-p-p38	1:1000 (WB)	Cell Signaling Technology	# 9211
Rabbit-anti-p38	1:1000 (WB)	Cell Signaling Technology	# 9212
Rabbit-anti-α-Tubulin	1:1000 (WB)	Cell Signaling Technology	# 2144
Rabbit-anti-GAPDH	1:1000 (WB)	Cell Signaling Technology	# 2118
Anti-Mouse IgG2b AF488	1:1000 (IF)	Invitrogen	# A21141
Anti-Rabbit IgG	1:2000 (WB)	Cell Signaling Technology	# 7074S
Anti-Mouse IgG	1:2000 (WB)	Cell Signaling Technology	# 7076S

Table S2. Sequence of the primers used for qRT-PCR assay.

Primer name	Sequence (5'-3')
KI67_F	CTGACCCTGATGAGAGTGAGGGA
KI67_R	TCTCCCCTTTTGAGAGGCGT
CCND1_F	GAGGCGGAGGAGAACAAACA
CCND1_R	GGAGGGCGGATTGGAAATGA
CCND2_F	AGCTGTCTCTGATCCGCAAG
CCND2_R	TGCTCCCACACTTCCAGTTG
EGR1_F	CACCTGACCGCAGAGTCTTT
EGR1_R	CTGACCAAGCTGAAGAGGGG
EGFR_F	GACAGGCCACCTCGTCG
EGFR_R	CCGGCTCTCCCGATCAATAC
SPRY1_F	TCCCTGCCCTGGATAAGGAA
SPRY1_R	CACGGCCGAAATGCCTAATG
TGFBR2_F	CTCCTGTGCAGCTTCCCTC
TGFBR2_R	GATGTGCGGGCCAGATGT
ILK1_F	TCCCTGGATCACTCCACAGT
ILK1_R	TCGTTCTCCGTGTTGTCCAG
